# Understanding your operating microscope's assistant scope and beamsplitter

**Published:** 2014

**Authors:** Ismael Cordero

**Affiliations:** Clinical Engineer, Philadelphia, USA. ismaelcordero@me.com

**Figure:**
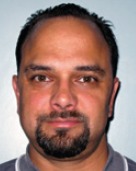
Ismael Cordero

In a previous issue (*Community Eye Health Journal* Vol 27 Issue 85) we reviewed ophthalmic operating microscopes. We learnt that they often include a second set of binoculars, part of what is commonly called an assistant or teaching scope, which allows another person to view the operation at the same time as the surgeon in charge.

**Figure:**
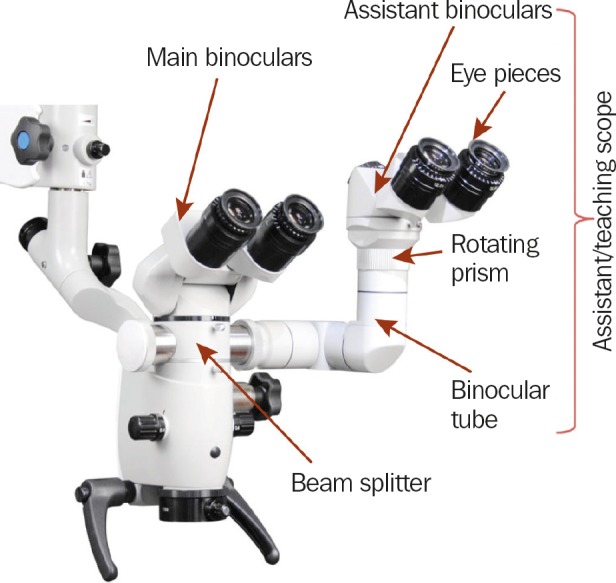
Figure 1

This is made possible by the beam splitter, which connects the assistant scope to the main visual path of the operating microscope. The optical components of the assistant scope (Figure 1) are almost identical to the main scope and consist of either fixed or inclinable binoculars. These have adjustable eyepieces for users with refractive error and a stereo observation tube that makes it possible to adjust the binoculars to a position comfortable for the assistant surgeon or trainee.

**Figure:**
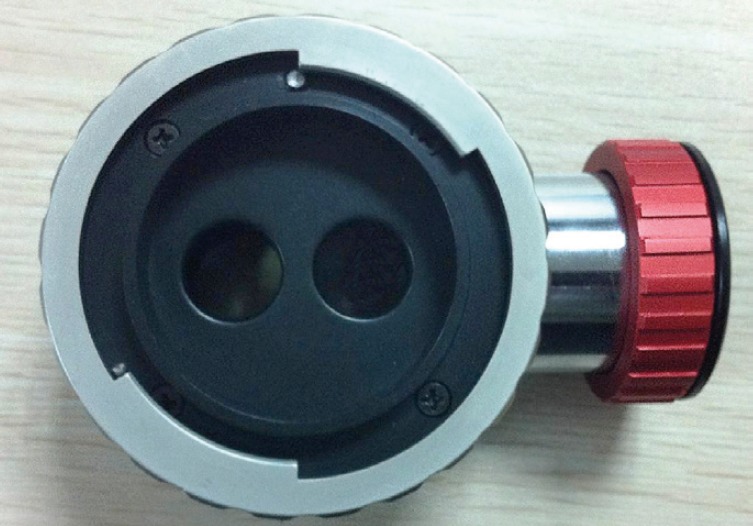
Figure 2a

In modern microscopes, the magnification and focus of the assistant scope match those of the main scope and are controlled by the surgeon in charge, using the foot pedal.

**Figure:**
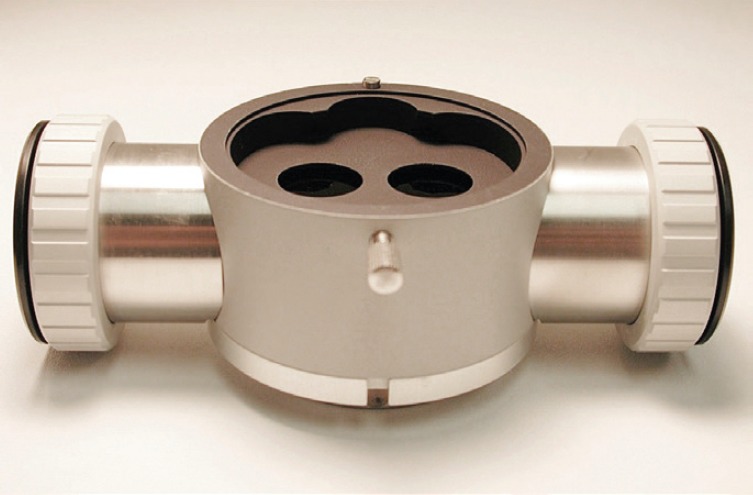
Figure 2b

The assistant scope has a rotating prism that allows the observer to orient the field of view. For **observation or teaching**, the field of view of the assistant scope coincides with that of the main scope, used by the surgeon. For example, if the surgeon says ‘See the spot at 3 o'clock’, the observer must also see it at 3 o'clock. For **an assistant surgeon**, the field of view of the assistant scope must be oriented to match the assistant's own position. The position of 3 o'clock as seen by the main surgeon would thus be at the 6 o'clock or 12 o'clock position for the assistant, depending on whether she/he is located to the right or left of the main surgeon.

**Figure:**
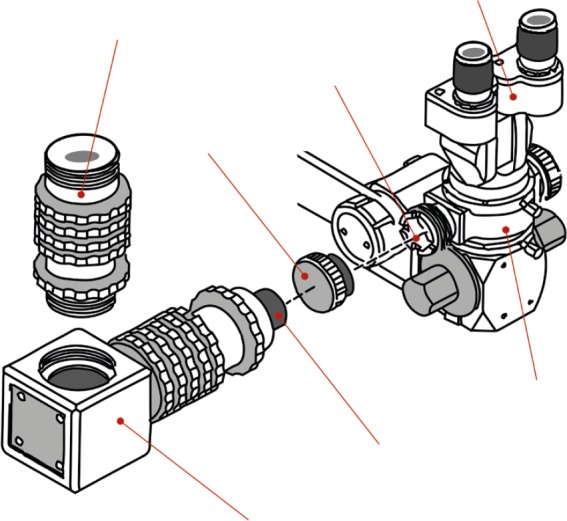
Figure 3

The beam splitter splits the light path to allow a video camera, digital camera, or an assistant scope to be attached to the microscope. Beam splitters can have one port, or adapter (Figure 2a), or two ports (Figure 2b).

Each beam splitter has a specific split ratio such as 50:50 or 70:30. The split ratio is marked on the body of the beamsplitter.

In the case of a 50:50 beam splitter, the amount of light is split equally between the main binoculars and the attachment(s), which is what is needed when an assistant scope is used.

A 70:30 beam splitter is used for photography and video. In this case, 70% of the light is directed to the main binoculars while the other 30% is directed to the attachment where the camera is connected.

The assistant scope, camera and other attachments connect to the beam splitter by means of a coupler that is made to fit the port of a particular model of beam splitter (Figure 3).

## Useful tips

- It is important to note that beam splitters, assistant scopes and other attachments are made for specific models of operating microscopes. For instance, if you have a Zeiss model microscope you will need a compatible Zeis model beam splitter and assistant scope. Many beam splitters and other attachments made by one manufacturer will not work on other brands of microscopes.
- You may need to adjust the balance and tension settings of the microscope suspension arm following the addition of beam splitters, assistant scopes, cameras and other accessories. Also, the surgeons may need to get used to the weight and balance of the additional equipment. There is a limited amount of weight a suspension arm can effectively hold. This weight is normally labeled on the suspension arm and, if not, this information can be found in the microscope's user manual.
- If you are considering obtaining an operating microscope for microsurgical training, it is an absolute necessity to have an assistant or teaching scope. Without it, microsurgical training cannot be effective.
- You can find refurbished assistant scopes and beam splitters that cost much less than new units.
- If you are not planning to use an assistant scope or camera, or to video record the operation, it is always good to remove the beam splitter and assistant microscope, camera or other attachment so that the image brightness is better.

